# The Height-Adaptive Parameterized Step Length Measurement Method and Experiment Based on Motion Parameters

**DOI:** 10.3390/s18041039

**Published:** 2018-03-30

**Authors:** Yanshun Zhang, Yingyue Li, Chuang Peng, Dong Mou, Ming Li, Wei Wang

**Affiliations:** 1 School of Instrumentation and Optoelectronic Engineering, Beihang University, Beijing 100191, China; zhangyanshun@buaa.edu.cn (Y.Z.); pengchuang@buaa.edu.cn (C.P.); liliyalm@buaa.edu.cn (M.L.); wangwei_b10@163.com (W.W.); 2 Electronic Engineering Research Institute, China Academy of Engineering Physics, Mianyang 621900, China; md1015@126.com

**Keywords:** motion parameters, gait analysis, step length, self-adaptation

## Abstract

In order to tackle the inaccurate step length measurement of people with different heights and in different motion states, a height-adaptive method of step length measurement based on motion parameters is proposed in this paper. This method takes people’s height, stride frequency, and changing accelerometer output while walking into integrated consideration, and builds a dynamic and parameterized model of their step length. In this study, these parameters were calibrated with thirty sets of experiment data from people with different heights and in different motion states, which were then verified experimentally by motion data of randomly selected subjects, regardless of speed and height. The experiment results indicate that the height-adaptive step length measurement was realized, thus eliminating the influence of height exerted on step length measurement.

## 1. Introduction

As one of the important parameters reflecting people’s motion characteristics, step length can be used in the research of measurements of body motion parameters, disease diagnosis and treatment, health monitoring, rehabilitation training, and pedestrian navigation [1,2,3,4,5]. Motion parameters measured by small Micro Electro Mechanical Systems (MEMS) inertial sensors, at a low cost and with high precision, render step length measurement feasible and effective [3,6].

Step length measurement bas been an important aspect of gait analysis. Many authors employ foot-mounted or leg-mounted IMU which is the abbreviation of “Inertial measurement unit” and can be used as measuring device to get data. However, this can cause a change in angle of the IMU, because of the transformation of the foot or leg when the subjects walk. We employed awaist-mounted IMU to measure the data, so that no matter how the angle of the foot changed, the orientation of the IMU would stay the same; this way, we were able to reduce the parameters of the algorithm [7,8,9,10].

Different body characteristics and motion parameters lead to unavoidable errors in step length measurement. This problem can be solved by adopting the corresponding step length measurement under different circumstances. At present, there are three main methods of step length measurement:

The first method calculates the step length based on geometry models. Cavagna et al. (among other scholars) believe that the displacement of the horizontal direction in a single foot motion during walking can be described as an inverted pendulum model, providing the mathematic relationship between the displacement of body’s center of gravity in the horizontal direction, and that in the vertical direction [11,12,13]. A modified pendulum model is proposed by González et al. to estimate real-time step length, with each step divided into the single foot support phase and the double foot support phase [14]. Without the prophase training, Kun-Chan Lan et al. also bring up a step length measurement method based on the Pythagorean theorem [15]. However, because of the integral operation involved, the methods based on geometry models mentioned above can easily result in drift errors.

The second method resorts to the nonlinear empirical formula of the step length. By studying human walking, Weinberg proposes the nonlinear step length measurement method based on the peak values and the valley values of the acceleration in the center of gravity [16]. Due to its simplicity and easy application, this formula is used by a group of scholars researching pedestrian navigation, either directly or indirectly [3,5,17,18]. Parameter recalibration is required when dealing with a variety of pedestrians.

The third method is based on the linear combination: Levi et al. utilize a constant and stride frequency [19], while Ladetto takes both stride frequency and acceleration variance into consideration [2,20,21]. If applied in pedestrians with different heights, these two methods require parameter recalibration, which lacks wide adaptability. Another linear combination method based on height and stride frequency is presented by Renaudin et al., with the measurement precision of step length increased [22]. The measurement precision, however, deteriorates when there are strenuous motions during human walking.

Summarizing advantages and disadvantages of the methods mentioned above, this paper introduces a novel step length measurement method with the integration of pedestrians’ height, stride frequency, and acceleration variance during walking, by analyzing the gait characteristics. Meanwhile, the corresponding experimental research is performed.

## 2. Analysis of Gait Characteristics

**Figure 1:**
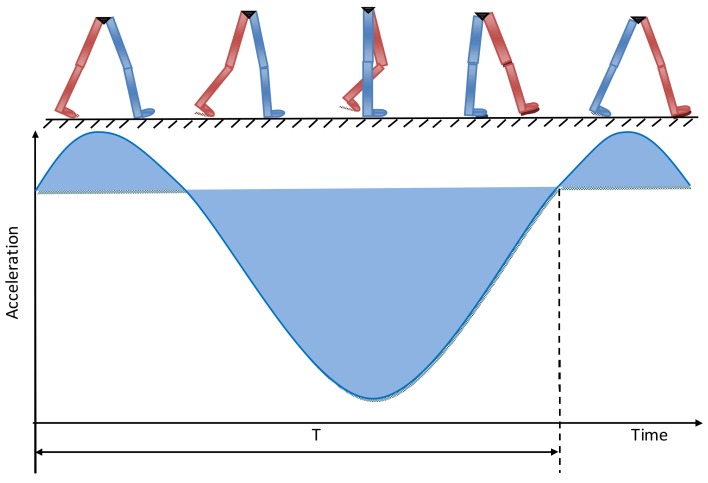
The relationship between the vertical acceleration and gait.

When people walk, there is a motion in virtually each part of body (feet, legs, waist, etc.) [23,24], which means the acceleration speed and angular velocity are constantly changing. The motion of feet and legs are relatively strenuous, with apparent acceleration and angular velocity change, making it easy to extract useful information from them. It is convenient to wear and fix the sensors at the waist, and there is only a little influence exerted on the body motion because of the gentle waist motion. When wearing sensors at the waist, the change of the acceleration and angular velocity in the vertical direction is more obvious than that in other directions, thus facilitating the analysis, extraction, and estimation of the human motion status. The vertical waist acceleration is shown in Figure 1.

**Figure 2:**
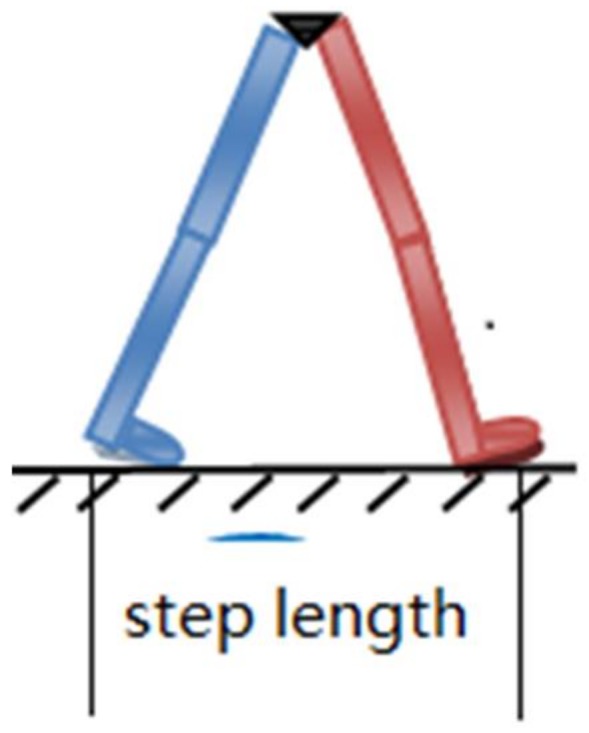
The illustration of the step length.

From the figure above, it is seen that the center of gravity changes periodically up and down with each step. In addition, the vertical acceleration of the center of gravity changes periodically too, which leads to a different step length resulting from different walking habits, body characteristics, and walking status. Here, “step length” is the distance between the footsteps of the left and right foot. As shown in Figure 2, the step length is the distance between the blue foot and the red foot. The relationship between the step length and the changing vertical acceleration needs to be studied. The corresponding parameters have to be identified and extracted from the accelerometer or gyroscope for real-time calculation.

**Figure 3:**
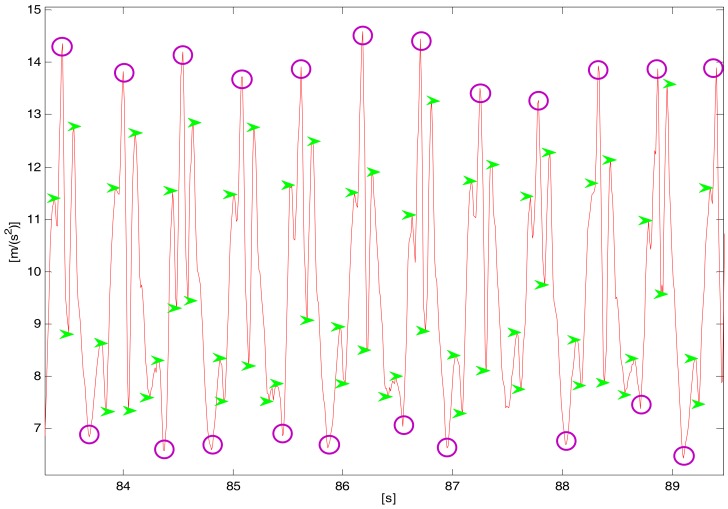
The change of vertical acceleration during walking.

In practical applications, as shown in Figure 3, there are sensor noises, different periodical peak values, and false peak values from the accelerator output resulting from sensor detection errors and step inconformity.

The overlay of walking speed, the periodical change of center of gravity, and the variation in the heaviness of step generates the vertical acceleration. All of these factors reflect walking status from which the step length is calculated. It is feasible to study the relationship between these factors and the step length, and then conduct the step length measurement.

## 3. Step Length Measurement Method

### 3.1. The Step Length Measurement Method Based on Stride Frequency and Acceleration Variance

By analyzing different walking speeds and types of gait per person during walking, Ladetto proposes the step length measurement based on the linear relation of the step length, stride frequency, and acceleration variance [18], which can be expressed as follows:

(1) $$SL_{i}=A\cdot f_{i}+B\cdot var_{i}+C$$

where *A* is the coefficient of the stride frequency, *B* is the coefficient of the vertical acceleration variance, and *C* is a constant. *A*, *B*, *C* are parameters calculated by the least square method, which is a form of mathematical optimization technology. It finds the best function match of the data by minimizing the sum of the square of the error; $f_{i}$ stands for the stride frequency, indicating how fast the pedestrian walks; var represents the acceleration variance during walking, describing whether the step is heavy or light: it can be calculated by Equation (4). These two factors indicate the pedestrian’s step length indirectly. *SL* is the short name of Step Length. In the positioning of the same walking person, this model is frequently applied in a precise manner [22,23,24]. However, when the application extends to different people, a lack of consideration about differences between individuals deteriorates the accuracy of the step length measurement.

### 3.2. The Step Length Measurement Method Based on Height, Stride Frequency and Acceleration Variance

For pedestrians with different heights, it is found that the step length is also different even when $f_{i}$ and $var_{i}$ are the same. According to the method mentioned in Section 3.1, when applied to different people, calibration is once again required for each individual to improve measurement accuracy, which limits its application. According to the kinetics of the human body, the step length is proportional to the leg length as well as the body height under normal circumstances.

By analyzing the research and methods described in Section 3.1, a novel step length measurement model based on height, stride frequency, and acceleration variance is proposed:

(2) $$SL_{i}=h\cdot (A\cdot f_{i}+B\cdot var_{i}+C)+D$$

where *i* represents the *i*th step during walking; *h*, $f_{i}$ and $var_{i}$ stand for height, stride frequency, and vertical acceleration variance during the *i*th step, respectively. A,B,C,D are the corresponding model coefficients.

Inputs of this method are height *h*, stride frequency $f_{i}$, and acceleration variance $var_{i}$. *h* is a fixed constant and remains unchanged during different walking processes of the same individual. $f_{i}$ can be obtained by Equation (3):

(3) $$f_{i}=\frac{1}{t_{i}-t_{i-1}}$$

where $t_{i}$ and $t_{i-1}$ represent the corresponding moments when detecting two adjacent steps [25]. In addition, the vertical acceleration variance of each step during walking can be calculated by Equation (4):

(4) $$var_{i}=\frac{1}{N-1}\sum_{t=t_{i-1}}^{t_{i}}{\left(a_{t}-{\bar{a}}_{i}\right)}^{2}$$

where $a_{t},{\bar{a}}_{i},N$ stand for the acceleration at the moment of *t*, the average acceleration, and the number of sampling points within one step, respectively.

According to Equations (2)–(4), the step length measurement is realized. In Figure 3, the flow chart of the step length measurement based on the low-cost MEMS inertial system is presented.

**Figure 4:**
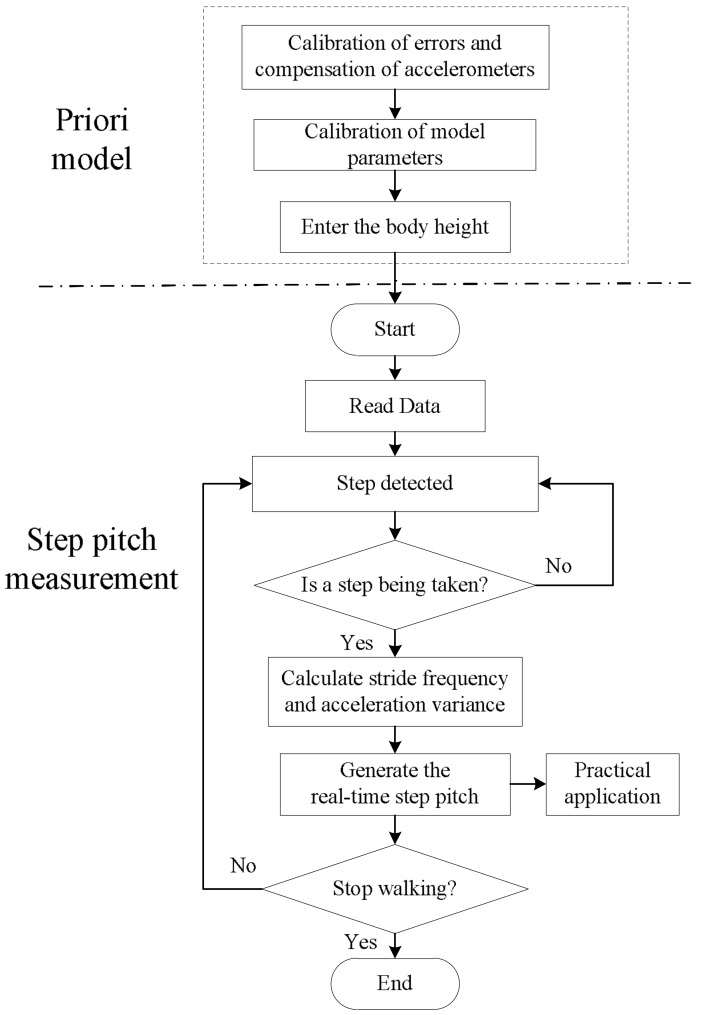
The flow chart of the step length measurement.

As shown in Figure 4, before calculating step length, errors of inertial sensors and parameters of the step length model are calibrated by the method presented in the reference paper [26]. Once the height of an individual is entered, the height-adaptive step length is calculated according to the program. Once calculations begin, data is read, and then the number of steps is detected. For every single step, the sensors calculate stride frequency $f_{i}$, acceleration variance $var_{i}$, and the step length *SL_i_*. *SL_i_* serves as the input to the practical application.

## 4. Experiment Research and Analysis

In order to verify the proposed method in this paper, the following experiments have been conducted. The experiments consist of two parts:

(1) The model parameter calibration of the height-adaptive and parameterized step length model;
(2) An evaluation of the accuracy of the step length measurement based on a walking experiment.

As a consequence of the experimental complexity and the unavailability of a high-speed synchronous camera shooting, the step length was measured, and its accuracy was verified indirectly, by walking along one fixed route several times over.

### 4.1. Experimental Equipment

**Figure 5:**
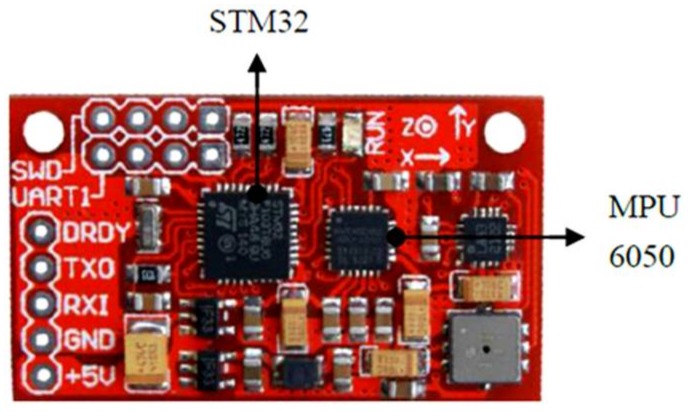
The signal collection and transmission module.

**Figure 6:**
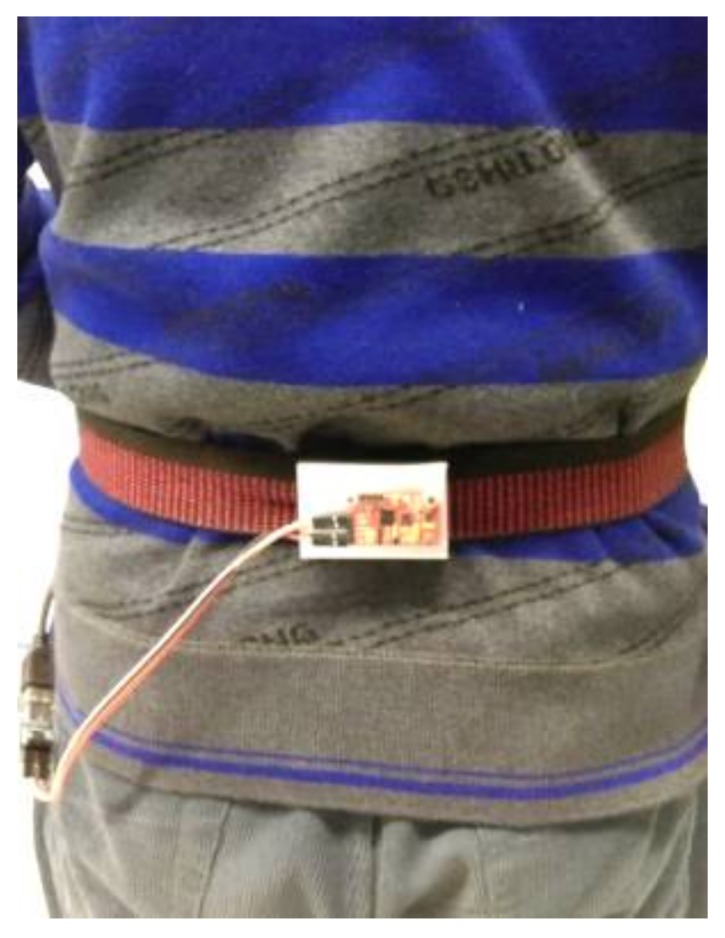
The wearing method.

The experimental equipment consisted of a signal acquisition and transmission module, a laptop, and a wearable device. As shown in Figure 5, the acceleration output of MPU6050 is acquired by the microprocessor STM32 in the signal acquisition and transmission module, which is then sent to the laptop through a serial interface. Once the raw data is received, the step length is calculated, and the DR navigation is conducted. DR is the abbreviation of “Dead reckoning”. Dead reckoning algorithm uses inertial navigation algorithm to predict motion position MPU-6050 is a MEMS inertial sensor with a 3-axis accelerometer and a 3-axis gyroscope, the advantages of which are its small size, its low cost, and its high precision. MEMS (Micro-Electro-Mechanical System) is also called micro-electromechanical system, micro-system, micro-mechanics, etc. It refers to high-tech devices with a size of a few millimeters or even less. The accelerometer features a measurement range of ±2 g, a zero bias of 50 mg, and random error of 0.4 mg. The random error of the gyroscope is due to the random variation of gyroscope output, which changes with time. It is expressed by the mean square error of the output data during idle state. As for the gyroscope, the measurement range is ±1000°/s, the zero bias is 0.4°/s, and the random error is 0.05°/s. The wearable device is tied up at the waist, as Figure 6 demonstrates.

### 4.2. Calibration of Experiment Parameters for the Step Length Model

**Figure 7:**
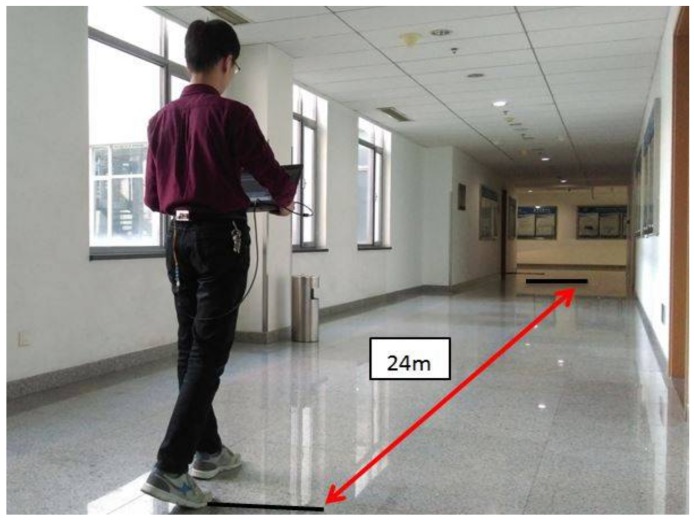
The parameter calibration experiment.

Before measuring step length using Equation (2), the parameters A,B,C,D need to be calibrated. The height was entered and the result was calculated. For more accurate calibrated parameters, the subjects of different heights such as 1.60 m, 1.63 m, 1.71 m, 1.78 m and 1.83 m were selected to walk along one fixed route at different speeds. As shown in Figure 7, each subject was asked to walk a certain distance (24 m) along a flat road at a slow speed, at a preferred speed and at a fast speed. Each trajectory was conducted twice. In total, there are 30 sets of data in Table 1. The walking speed during each set of the experiment was made to be as stable and as consistent as possible. In order to reduce random errors, the average step length, the stride frequency and the vertical acceleration variance for one step were measured and calculated. Besides the walking data, the subject’s height is also added, thus enhancing the experimental data to include different heights, different step frequencies, different variances, and different step lengths. Finally, the model parameters of the step length measurement proposed in this paper were calibrated by the least square method. The variable was written as *a* = $\left[\begin{array}{cc} \begin{array}{c} 1\\ 1 \end{array} & \begin{array}{c} \begin{array}{cc} f_{1} & \begin{array}{cc} var_{1} & h_{1} \end{array} \end{array}\\ \begin{array}{cc} f_{2} & \begin{array}{cc} var_{2} & h_{2} \end{array} \end{array} \end{array}\\ 1 & \begin{array}{cc} f_{3} & \begin{array}{cc} var_{3} & h_{3} \end{array} \end{array} \end{array}\right]$, the coefficient as *b* = $\left[\begin{array}{c} \begin{array}{c} A\\ B \end{array}\\ \begin{array}{c} C\\ D \end{array} \end{array}\right]$, and the *SL* as *SL* = $\left[\begin{array}{c} {SL}_{1}\\ {SL}_{2}\\ {SL}_{3} \end{array}\right]$. Then the formula was minimized to ${\left||ab-SL|\right|}_{2}$, in order to calibrate the coefficient *A*, *B*, *C*, *D*.

The step length model in Section 3.1 has nothing to do with height. For a better comparison and analysis, the experimental data of the subject with the height of 1.71 m was calibrated.

### 4.3. Walking Experiments

During walking, the pedestrian’s step length is not technically consistent for each step, leading to the unobtainability of precise measurements. Consequently, walking experiments of a certain distance are performed to verify the feasibility and accuracy of the step length measurement method proposed in this paper. Three other subjects were chosen to walk along the standard track in the playground of Beihang University three times over, from which the mean value was calculated. The experiment was conducted as follows: (1) the signal acquisition and transmission module was attached to the subject’s waist with a belt; (2) the walking experiment was conducted and the motion data was acquired during walking; (3) the step length was measured by both the method explored in this paper and the method based on stride frequency and variance separately; (4) the experiment data was analyzed, and the results of the two different methods were compared.

The step length during walking was summed up to obtain the total walking distance, which was compared with the actual path length. The number of steps, the mean step length, the total walking distance, and the error rate are listed in Table 2.

The actual path length is the length of the track, namely, 400 m, but the length of the track of a few groups ended up being 453 m, because there were students taking physical education class when we were conducting our experiment. Therefore, the subjects were walking on the playground’s outer ring, the actual path length of the track thus being 453 m. The walking distance was calculated based on the estimated step length in real time.

Experimental results indicate that the precision of the step length measurement of the method proposed in this paper is superior to that of the method based on stride frequency and variance. By comparing the average error and the standard deviation, we can conclude that the method in this paper can be used for subjects with different heights, the error is more constant than the method based on the frequency and acceleration variance during walking. More specifically, when adopting the step length measurement method based on stride frequency and variance, the user has to be the same person or at least someone with similar physical characteristics. A calibration of parameters had to be conducted again to achieve favorable results for different users, thus restricting its wide application. In this paper, the body height is added to the proposed step length measurement model. Despite the different heights of users, the step length measurement accuracy is relatively high.

## 5. Conclusions

This paper proposes a height-adaptive step length measurement method based on the low-cost MEMS inertial system. Taking the height, the stride frequency, and the vertical acceleration variance into account, the step length was estimated with the motion data measured by the output of the accelerometer worn at the pedestrian’s waist. Without any parameter calibration, this method is highly height-adaptive, that is to say, different users just need to input different heights for the step length to be properly measured. In addition, a series of walking experiments were performed, the results of which prove that this method can measure the step length accurately, giving rise to a great application prospect in fields such as auxiliary medical treatment, exercise rehabilitation, and more.

## Figures and Tables

**Table 1:** The data for parameter calibration.

Groups	Height (m)	Stride Frequency m (Hz)	Acceleration Variance	Step Length (m)
1	1.60	1.6418	0.1662	0.5455
2	1.60	1.5842	0.1253	0.5217
3	1.60	1.8242	0.4685	0.6154
4	1.60	1.8470	0.3424	0.6000
5	1.60	1.8377	0.5645	0.6316
6	1.60	1.9035	0.8991	0.6857
7	1.63	1.5820	0.2578	0.6154
8	1.63	1.5016	0.2138	0.6000
9	1.63	1.7242	0.6021	0.7102
10	1.63	1.7566	0.5718	0.7059
11	1.63	1.8346	1.2402	0.8000
12	1.63	1.8831	1.1853	0.8000
13	1.71	1.7640	0.5182	0.6667
14	1.71	1.7624	0.3912	0.6667
15	1.71	1.8278	0.9619	0.7273
16	1.71	1.8471	0.8618	0.7273
17	1.71	1.9942	1.4286	0.8000
18	1.71	2.0901	1.8048	0.8276
19	1.78	1.4914	0.2503	0.6667
20	1.78	1.6307	0.3343	0.7059
21	1.78	1.7328	0.5587	0.7500
22	1.78	1.7476	0.5065	0.7500
23	1.78	1.9508	1.2965	0.8889
24	1.78	1.9738	1.2014	0.8571
25	1.83	1.4564	0.2006	0.6486
26	1.83	1.6707	0.2367	0.6857
27	1.83	1.7334	0.4753	0.7500
28	1.83	1.7614	0.5075	0.7500
29	1.83	2.0869	1.2029	1.0000
30	1.83	2.0604	1.2742	0.9600

**Table 2:** The results of the walking experiments.

	Groups	Height	Step Number	Mean step Length (m)	Actual Path Length (m)	Walking Distance (m)	Error Rate (%)	Average Error (%)	Standard Deviation
The method based on *f* and var	1	155	638	0.7408	400	472.6304	18.16	7.904	5.8297
	2	158	820	0.6933	453	568.4709	25.49		
	3	160	636	0.7699	453	489.6721	8.09		
	4	161	634	0.7868	453	498.8594	10.12		
	5	162	616	0.7058	400	434.7728	8.69		
	6	163	659	0.6811	400	448.8564	12.21		
	7	164	501	0.7119	400	356.6674	10.83		
	8	165	578	0.7027	400	406.1556	1.53		
	9	166	607	0.6845	400	415.5034	3.87		
	10	167	615	0.6894	400	423.9888	5.99		
	11	168	572	0.6736	400	385.2728	3.68		
	12	170	636	0.6922	453	440.2300	2.82		
	13	171	513	0.7317	400	375.3375	6.17		
	14	172	532	0.7284	400	387.5195	3.12		
	15	173	482	0.7451	400	359.1375	10.21		
	16	175	567	0.6879	400	390.0232	2.49		
	17	177	475	0.7956	400	377.9307	5.52		
	18	180	549	0.7477	400	410.4711	2.62		
	19	181	540	0.6722	400	366.7747	8.31		
	20	184	618	0.6732	453	416.0329	8.16		
The proposed method	1	155	638	0.6385	400	407.5732	1.89	2.2215	1.3088
	2	158	820	0.5649	453	463.2410	2.26		
	3	160	636	0.6866	453	436.7043	3.59		
	4	161	634	0.7404	453	469.4238	3.63		
	5	162	616	0.6455	400	397.6452	0.59		
	6	163	659	0.6175	400	406.9507	1.74		
	7	164	501	0.7762	400	388.8873	2.78		
	8	165	578	0.6790	400	392.4701	1.88		
	9	166	607	0.6540	400	397.0079	0.75		
	10	167	615	0.6714	400	412.9192	3.23		
	11	168	572	0.6581	400	376.4516	5.88		
	12	170	636	0.6154	400	395.0054	1.25		
	13	171	513	0.7640	400	391.9444	2.01		
	14	172	532	0.7577	400	403.0730	0.77		
	15	173	482	0.7989	400	385.0925	3.73		
	16	175	567	0.6949	400	394.0304	1.49		
	17	177	475	0.8211	400	390.0489	2.49		
	18	180	549	0.8358	453	458.8721	1.3		
	19	181	540	0.7236	400	390.7405	2.31		
	20	184	618	0.7267	453	449.0835	0.86		

## References

[1] Durie N.D., Farley R.L. (1980). An apparatus for step length measurement. J. Biomed. Eng..

[2] Shin S.H., Chan G.P. (2011). Adaptive step length estimation algorithm using optimal parameters and movement status awareness. Med. Eng. Phys..

[3] Sayeed T., Samã A., Catalã A., Rodríguez-Molinero A., Cabestany J. (2015). Adapted step length estimators for patients with Parkinson’s disease using a lateral belt worn accelerometer. Technol. Health Care Off. J. Eur. Soc. Eng. Med..

[4] Juen J., Cheng Q., Schatz B. (2015). A natural walking monitor for pulmonary patients using mobile phones. IEEE J. Biomed. Health Inform..

[5] Zhuang Y., Lan H., Li Y., El-Sheimy N. (2015). PDR/INS/WiFi integration based on handheld devices for indoor pedestrian navigation. Micromachines.

[6] Renaudin V., Combettes C. (2014). Magnetic, Acceleration Fields and Gyroscope Quaternion (MAGYQ)-Based Attitude Estimation with Smartphone Sensors for Indoor Pedestrian Navigation. Sensors.

[7] Müller P., Seel T., Schauer T. Experimental Evaluation of a Novel Inertial Sensor Based Realtime Gait Phase Detection Algorithm. Proceedings of the European Conference on Technically Assisted Rehabilitation—TAR 2015.

[8] Gao Y., Jiang Z., Ni W., Vasic Z.L., Cifrek M., Du M., Vai M.I., Pun S.H. (2017). A Novel Gait Detection Algorithm Based on Wireless Inertial Sensors. CMBEBIH 2017.

[9] Seel T., Graurock D., Schauer T. (2015). Realtime assessment of foot orientation by accelerometers and gyroscopes. Curr. Dir. Biomed. Eng..

[10] Brahms C.M., Zhao Y., Gerhard D., Barden J.M. (2018). Stride length determination during overground running using a single foot-mounted inertial measurement unit. J. Biomech..

[11] Cavagna G.A., Thys H., Zamboni A. (1976). The sources of external work in level walking and running. J. Physiol. (Lond.).

[12] Zijlstra W., Hof A. (2003). Assessment of spatio-temporal gait parameters from trunk accelerations during human walking. Gait Posture.

[13] Brandes M., Zijlstra W., Heikens S., van Lummel R., Rosenbaum D. (2006). Accelerometry based assessment of gait parameters in children. Gait Posture.

[14] González R.C., Alvarez D., López A.M., Alvarez J.C. Modified pendulum model for mean step length estimation. Proceedings of the International Conference of the IEEE Engineering in Medicine and Biology Society (EMBS 2007).

[15] Lan K.C., Shih W.Y. (2014). Using smart-phones and floor plans for indoor location tracking. IEEE Trans. Hum. Mach. Syst..

[16] Weinberg H. Using the ADXL202 in Pedometer and Personal Navigation Applications. Analog Devices AN-602 Application Note.

[17] Ho N.H., Truong P.H., Jeong G.M. (2016). Step-Detection and Adaptive Step-Length Estimation for Pedestrian Dead-Reckoning at Various Walking Speeds Using a Smartphone. Sensors.

[18] Zhu Y., Zhang R., Xia W., Jia Z., Shen L. A hybrid step model and new azimuth estimation method for pedestrian dead reckoning. Proceedings of the Sixth International Conference on Wireless Communications and Signal Processing.

[19] Levi R.W., Judd T. (1996). Dead Reckoning Navigational System Using Accelerometer to Measure Foot Impacts. U.S. Patent.

[20] Ladetto Q. On Foot Navigation: Continuous Step Calibration Using Both Complementary Recursive Prediction and Adaptive Kalman Filtering. Proceedings of the ION GPS, Salt Lake City.

[21] Qian J., Pei L., Zou D., Qian K., Liu P. Optical flow based step length estimation for indoor pedestrian navigation on a smartphone. Proceedings of the Position, Location and Navigation Symposium (PLANS 2014).

[22] Renaudin V., Susi M., Lachapelle G. (2012). Step length estimation using handheld inertial sensors. Sensors.

[23] Shibuya N., Nukala B.T., Rodriguez A.I., Tsay J., Nguyen T.Q., Zupancic S., Lie D.Y. A real-time fall detection system using a wearable gait analysis sensor and a Support Vector Machine (SVM) classifier. Proceedings of the Eighth International Conference on Mobile Computing and Ubiquitous Networking.

[24] Kauw-A-Tjoe R., Thalen J., Marin-Perianu M., Havinga P. SensorShoe: Mobile Gait Analysis for Parkinson’s Disease Patients. Proceedings of the UbiComp 2007 Workshops, University of Innsbruck.

[25] Zhang Y., Xiong Y., Wang Y., Li C., Wang Z. (2016). An Adaptive Dual-Window Step Detection Method for a Waist-Worn Inertial Navigation System. J. Navig..

[26] Zhang Y., Xu Y., Xing X., Wang Z., Xiong Y. (2016). The Standing Calibration Method of MEMS Gyro Bias for Autonomous Pedestrian Navigation System. J. Navig..

